# Periprosthetic seromas and a third space effect after high-dose methotrexate

**DOI:** 10.1007/s00508-024-02467-6

**Published:** 2024-11-11

**Authors:** Claudia Prattes, Andreas Leithner, Joanna Szkandera, Georg Prattes, Ernst-Christian Urban, Andrea Eder-Halbedl, Volker Strenger

**Affiliations:** 1 Department of Orthopaedics and Trauma, Medical University of Graz, Auenbruggerplatz 5, 8036 Graz, Austria; 2 Department of Pediatrics and Adolescent Medicine, Medical University of Graz, Auenbruggerplatz 30, 8036 Graz, Austria; 3 Division of Clinical Oncology, Department of Internal Medicine, Medical University of Graz, Auenbruggerplatz 15, 8036 Graz, Austria; 4 Department of Anesthesiology, Medical University of Graz, Auenbruggerplatz 29, 8036 Graz, Austria

**Keywords:** Osteosarcoma treatment, HDMTX, Toxicity, Limb salvage surgery, Accumulation in cavities

## Abstract

**Background:**

Besides surgery, chemotherapy including high-dose methotrexate is a mainstay of osteosarcoma treatment. Methotrexate is known to accumulate in tissues and cavities, so-called third spaces (e.g., periprosthetic seromas) leading to local toxicity and delayed elimination (third space effect). We compared the concentrations of methotrexate in serum and periprosthetic seromas to evaluate a potential toxic risk based on a third space effect.

**Methods:**

In 45 osteosarcoma patients who were treated with endoprosthesis and high-dose methotrexate (HDMTX) between 1991 and 2011 we retrospectively analyzed methotrexate concentrations in periprosthetic seromas and serum. Differences were assessed by means of the Wilcoxon test.

**Results:**

A total of 112 periprosthetic seroma punctures were performed in 18 out of 45 patients. At 24 h the periprosthetic seroma concentrations were in median 14.86-fold (range 1.49–42.97-fold, *p* = 0.001), at 48 h in median 8.50-fold (range 1.36–52.56, *p* < 0.001) and at 72 h in median 2.66-fold (range 0.66–5.82, *p* = 0.015) of the corresponding serum concentrations. At 24 h highly toxic concentrations (≥ 20 μmol/l) were observed in 30% of all analyzed seromas (median 109.83 μmol/l, range 4.91–170.71 μmol/l). A significantly higher serum concentration (range 0.16–0.75 μmol/l, median 0.36 µmol/l) was found in patients with prior puncture than patients without puncture at 45 h after HDMTX.

**Conclusion:**

Methotrexate concentrations of periprosthetic seromas are significantly higher than corresponding serum concentrations possibly contributing to a third space effect. To avoid severe adverse effects punctures of these effusions should be considered.

## Introduction

While osteosarcomas are the most common primary malignancies of bones, which mainly occur in children, adolescents, and young adults (below 24 years) [1, 2], they are rare representing less than 1% of all malignant cancers diagnosed in the USA [3]. It is an aggressive disease with a 5-year event-free survival of 54% (95% confidence interval, CI 52–56%) in patients aged ≤40 years with M0 or M1 skeletal high-grade osteosarcoma [4]. Although collaboration of osteosarcoma study groups and the launch of adequate surgery and multi-agent chemotherapy improved the overall survival rates, there have been no further enhancements within the last two decades [1, 2, 5–10]. The dose intensification of methotrexate (MTX) is responsible for severe adverse effects, including toxicity concerning kidneys, liver, lungs, bone marrow, skin and mucosa [12–17]. It has been shown that the concentration as well as the duration of exposure of antineoplastic agents appear to be critical factors in the development of toxicity [11, 18–21]. Moreover, the administration of high-dose methotrexate (HDMTX) causes accumulation in tissues and cavities, the so-called third spaces, leading to local toxicity and delayed elimination [21–29].

Toxicity is based on MTX as a folic acid antagonist that inhibits dihydrofolate reductase. Normally this enzyme reduces dihydrofolate to its active form, tetrahydrofolate, which is an essential cofactor in DNA, purine and protein synthesis. The MTX binds the dihydrofolate reductase, blocks it from recycling and diminishes reduced folate pools [16, 17, 20, 22, 24, 30]. Finally, MTX induces cytotoxic cell damage in the S cell cycle phase [31, 32], therefore it is used as a widely effective anti-metabolite cancer treatment [14, 16, 17, 21, 30–32]. The kidneys are the principal route of excretion of HDMTX. Some studies claim that normal serum creatinine and a glomerular filtration rate >60 ml/min ensure sufficient clearance of HDMTX [15, 21, 25]. These criteria do not entirely nullify the risk of toxicity [14, 15, 33].

Doses of HDMTX would be fatal without supportive care measures [12–14, 20, 22, 31, 33]. These include the administration of leucovorin (LV, folic acid) together with adequate fluids, electrolytes and bicarbonates to maintain the urine output and to alkalinize the urine pH [14, 17, 20, 22, 30, 31, 33, 34]. Leucovorin rescue supplies the product of inhibited enzymes and is administered several hours after infusion. The preventive measures to reduce side effects of HDMTX include avoidance of drug interaction and drainage of third spaces [16, 21]. In order to minimize adverse effects, monitoring of MTX serum concentrations is routinely performed every 24 h after the start of the MTX infusion until the serum MTX level is considered to be safe. [14, 16, 20, 21, 24, 26, 28, 29, 33, 35].

Currently, there are different studies reporting about the influence of third spaces (also called malignant effusions, third space fluids or effusion spaces) on interindividual variations in the pharmacokinetics of MTX. According to these studies third spaces are responsible for a higher risk of developing toxicity, prolonged excretion and even for drug-related deaths [23, 26, 27, 29, 36].

To our knowledge for the first time we analyzed third spaces in terms of periprosthetic seromas in patients who were treated for osteosarcomas. The aim of our study was to evaluate MTX concentrations in periprosthetic seromas in comparison with corresponding MTX concentrations in serum to describe a possible contribution to a third space effect justifying punctures despite the increased risk of infections this carries.

## Patients, material and methods

We performed a monocentric, retrospective analysis of 59 consecutive patients, who had been treated with a limb salvage surgery with endoprosthesis and chemotherapy including HDMTX between 1991 and 2011 for histologically confirmed high-grade osteosarcoma at the Medical University of Graz. Of the cases 14 (31%) had to be excluded because of missing HDTMX data. Thus, 45 patients (28 male and 17 female patients) were analyzed. The median age at the time of diagnosis was 14.4 years (range 4.9–45.6 years). In these 45 patients we analyzed 514 episodes of HDMTX and 112 punctures.

The treatment was based on aggressive surgery and multi-agent chemotherapy according to the European and American Osteosarcoma Study (EURAMOS) group [37], the Cooperative Osteosarcoma Study group (COSS-96) [38], COSS-91 [39], COSS-86 [40] and EUROpean Bone Over 40 Sarcoma Study (EURO-BOSS) [41] regimens. According to these protocols HDMTX was provided intravenously 12 times with 12g/m^2^ over 4 h with an interval of at least 1 week. Supplementarily, all patients received an intravenous or oral leucovorin rescue (initial dose of 15 mg/m^2^ started 24–28 h after MTX infusion, later on according to the MTX serum level), hydration and alkalinization. We analyzed MTX serum concentrations, which were routinely determined 24, 48 and 72 h after starting the HDMTX infusion according to the respective protocols. Furthermore, serum drug monitoring was performed until the MTX concentration was <0.01 µmol/l. Delayed MTX elimination was treated with increased hydration and additional calcium folinate administration according to the treatment protocol. Additionally, in our institution significant periprosthetic seromas were punctured before and/or after administration of HDMTX to avoid third space effects. The seromas were diagnosed clinically and punctured under sterile conditions. The amount of punctured fluid was documented and the MTX concentrations of the periprosthetic seromas were determined, so these data were available for analysis. All laboratory values including drug MTX monitoring were determined at the Clinical Institution for Medical and Chemical Laboratory Diagnostic, Medical University of Graz. The MTX concentrations were measured by fluorescence polarization immunoassay technology in the same way for serum and seromas.

The MTX concentrations of serum and periprosthetic seromas were analyzed by descriptive statistical methods. In addition, we compared serum MTX concentrations routinely obtained 24, 48 and 72 h after end of the HDMTX infusion according to the respective treatment protocols with MTX concentrations of corresponding periprosthetic seromas obtained 6h before to 6h after the respective serum sample. Differences between concentrations of periprosthetic seromas and corresponding serum were assessed by means of the Wilcoxon test. Differences in serum concentrations between patients with and without prior puncture of a periprosthetic seroma in the respective treatment cycle were assessed by means of the Mann-Whitney U‑test. Statistical analyses were performed by using SPSS for Mac 20 (SPSS Inc., Chicago, Ill., USA). The study was approved by the Ethics Committee of the Medical University of Graz (EC-number 24-527 ex 11/12).

## Results

In 45 included patients (28 male and 17 female) in total 514 episodes of HDMTX were administered. For further details on patient characteristics see Table 1.

**Table 1:** Patient baseline characteristics: age, height, weight, body surface area (BSA), HDMTX dosage, creatinine at start of HDMTX, documented toxicities, osteosarcoma site, type and regression grade

**Baseline characteristics**	**Median (min-max)**	**Unit**
Age	14.4 (4.9–45.6)	Years
Height	168.0 (108.0–199.0)	cm
Weight	56.7 (16.9–99.5)	kg
BSA	1.65 (0.73–2.36)	m^2^
HDMTX dosage	19 (8.8–25)	g
Creatinine during therapy	0.71 (0.29–1.18)	mg/dl
**Documented toxicity**	**Absolute count (*N*)**	**Percentage (%)**
Fatigue	4	4
Anorexia	7	7
Deafness	8	8
Thrombocytopenia	14	14
Leukopenia	15	15
Mucositis	20	20
Nausea/vomiting	30	30
Delayed elimination	40	40
*Osteosarcoma site*		
Distal femur	29	64
Proximal tibia	12	27
Humerus	4	9
*Osteosarcoma subtype*		
Osteoblastic	13	29
Chondroblastic	10	22
Fibroblastic	4	9
Unknown	14	31
Histological type*		
Central, conventional high-grade osteosarcoma (G3–G4)	33	73
Unknown type	12	26
Regression grade 2	5	11
Regression grade 3	15	33
Regression grade 4	11	24
Regression grade 5	14	31

HDMTX dosage in g according to 12g/m^2^; regression grades defined by Salzer-Kuntschik [42]*Min*, minimum; *max*, maximum; *HDMTX* high-dose methotrexate; *N* number; *%* number in percentage

*histological type was assessed at definitive surgery

**Fig. 1:**
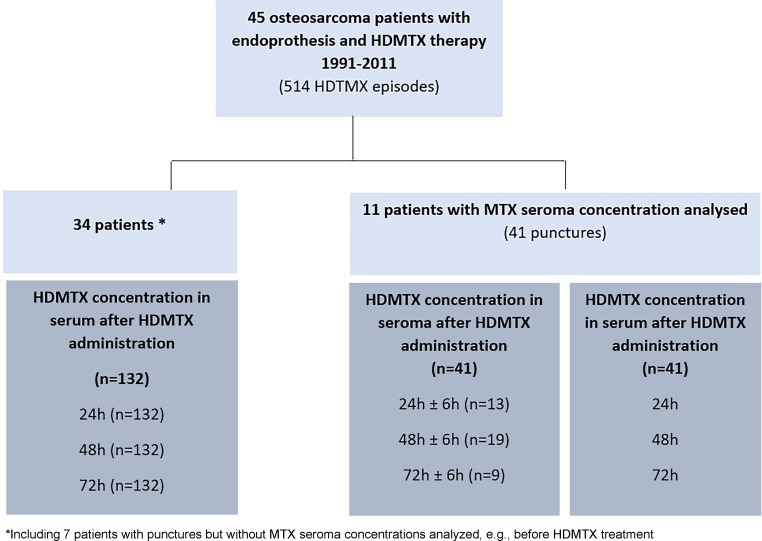
Flow-chart of analyzed patients

Periprosthetic seromas occurred in 18 patients (40%). In these patients 112 punctures were performed (range 1–20, median 5 punctures per patient). The volume of fluid from punctured periprosthetic seromas was documented in 101 punctures ranging from 5–420 ml (median 150 ml). The MTX concentrations were determined in 71 punctures performed in 11 patients. The interval to the corresponding serum HDMTX administration was available in 41 puncture measurements and was assigned to the corresponding serum concentrations (24, 48 or 72 h after HDMTX administration ±6 h) in all of them (Fig. 1).

**Fig. 2:**
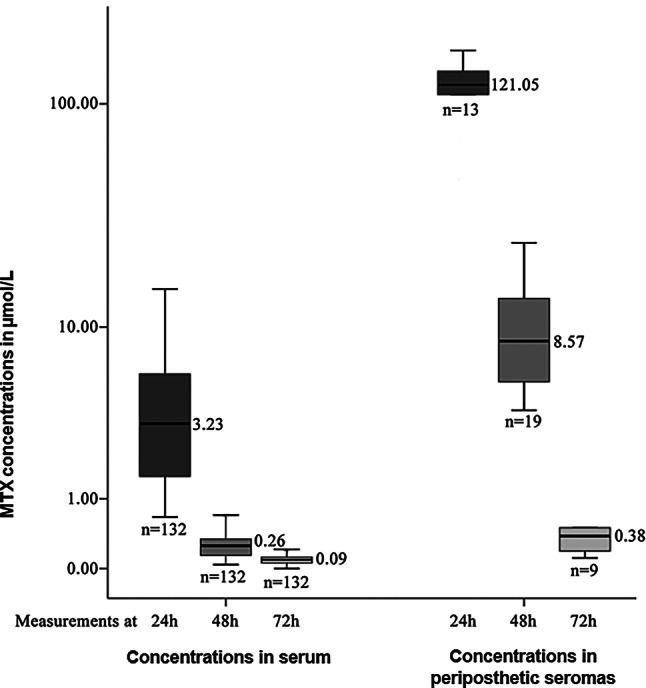
MTX concentrations in serum (*left*) and periprosthetic seromas (*right*) at 24, 48 and 72 h after HDMTX administration (in μmol/l)

The MTX concentrations in serum (*n* = 132 each, at 24, 48 and 72 h) and in periprosthetic seromas (*n* = 13 at 24 h, *n* = 19 at 48 h and *n* = 9 at 72 h) were within a wide range as shown in Fig. 2.

The comparison of both serum concentrations and periprosthetic seroma concentrations showed statistically significant differences between the three different time points (see Table 2). In 41 periprosthetic seroma punctures with available MTX concentrations these were significantly higher than the corresponding serum concentrations and ranged up to highly toxic levels (median 109.83 μmol/l, range 4.91–170.71 μmol/l at 24 h after HDMTX). Periprosthetic seroma concentrations were in median 35.54 times (range 3.79–13.27) higher than the corresponding serum MTX concentrations. For further details for different time points see Table 2.

**Table 2:** MTX concentrations (in μmol/L) measured in serum and periprosthetic seromas as well as ratios between serum and periprosthetic seroma concentrations

Time	C in serum	C in seromas	*p* value	Ratio
24 h	3.09 (0.37–45.0)	109.83 (4.91–170.74)	=0.001	35.54 (14.02–3.79)
48 h	0.26 (0.04–3.39)	2.64 (0.85–24.36)	<0.001	10.15 (21.25–7.18)
72 h	0.09 (0.01–1.21)	0.38 (0.11–2.5)	=0.015	4.22 (11.00–2.06)

At 24, 48 and 72 h from start of infusion: median (ranges), *p* values according to Wilcoxon test

*C* concentration

At 48 h after HDMTX patients who underwent prior puncture of a significant periprosthetic seroma (*n* = 41) had significantly higher serum concentrations (range 0.16–0.75 µmol/l, median 0.36 µmol/l) than patients without prior puncture (range 0.07–11.04 µmol/l, median 0.25 µmol/l Mann-Whitney U‑test, *p* = 0.045). At 24 and 72 h after HDMTX, there was no statistically significant difference of serum concentrations between patients with and without prior puncture of a periprosthetic seroma in the respective treatment cycle (Mann-Whitney U‑test, *p* = 0.57 at 24 h and *p* = 0.45 at 72 h).

## Discussion

In the present study we investigated MTX concentrations in osteosarcoma patients who received HDMTX following limb salvage surgery. Our results showed that MTX concentrations in periprosthetic seromas were significantly higher than in corresponding serum concentrations and reached highly toxic levels 24 h after HDMTX application. As such, periprosthetic seromas in osteosarcoma patients may act and should be considered as third spaces for high MTX levels.

In recent literature, similar results were found for third space compartments such as pleural effusions and ascites after HDMTX treatment. It has been postulated that MTX accumulation and the slow release into third space fluids result in a prolonged plasma MTX half-life, decreased clearance and thus, in an unpredictable toxicity [16, 27, 28]. In this respect the concentration and exposure duration of chemotherapeutic agents may influence the development of diverse toxic events [11, 12, 17–21]. Even though some researcher described a two-compartment model for the pharmacokinetic behavior of MTX, the present results indicate that MTX is not evenly distributed throughout the central and peripheral compartment. In accordance, Pauley et al. and Wan et al. mentioned a triphasic decline of MTX in their case reports [29, 42]. Anyhow, the reason for the slow release has been investigated in only few studies. Howell et al. pointed out that the rate of change in the third space is a function of the effusion volume, infusion rate and concentration between third space and serum resulting from the permeability and area of limiting membrane [32]. Other researchers hypothesized that a change in osmotic pressure and the placement of drug carrier complexes may be responsible for a slow release. As the protein content in cavities is similar to that in blood, chemotherapeutic agents may accumulate in such spaces and further influence plasma drug pharmacokinetics [28, 36].

Due to the consistently higher MTX level in seromas than in serum concentrations this study emphasizes to closely monitor osteosarcoma patients following HDMTX for severe toxicity. In addition, the present results should help to assess the potential indication of evacuating third space fluids prior to HDMTX administration as recommended in the existing literature [26, 27, 43, 44]. Anyhow, the potential indication of a puncture must be individually weighed against the increased risk of infections in these immunocompromised patients during chemotherapy. If a drainage is not possible for any reason, HDMTX dose should be at least reduced [45]. Moreover, our data show that puncture of significant periprosthetic seromas does not decrease the therapeutic level of MTX in serum in comparison to those without puncture. There was no statistically significant difference found in serum concentrations between patients with and without prior puncture of a periprosthetic seroma in the respective treatment cycle at 24 and 72 h. Interestingly, at 48 h after HDMTX patients with prior puncture had significant higher MTX serum levels than those without prior puncture. It is unlikely that punctures of periprosthetic seromas result in higher serum concentrations. It should rather be hypothesized that higher MTX serum concentrations facilitate the development of significant periprosthetic effusions or that significant periprosthetic seromas (and only those were punctured) lead to delayed MTX elimination even before puncture. After 2011 the incidence of postoperative seromas declined, possibly based on the introduction of a different type of megaprosthesis and modified reconstruction methods.

There are some limitations of our study, mostly arising from its retrospective design. Additionally, the lack of a standardized protocol for puncturing leading to different time points of serum sampling is a limiting factor of this study. Punctures were performed not exactly at the same time points as routine serum monitoring at 24, 48 and 72 h from the start of infusion but within a time frame of 6h before to 6h after serum monitoring. Anyhow, in light of the highly significant differences this does not weaken the overall conclusion of our analyses. Moreover, all patients with significant seromas were routinely punctured at the discretion of the treating physicians. Thus, we were not able to reliably evaluate the clinical effect of this procedure. A comparison of MTX kinetics, toxicities or treatment outcomes is only feasible between episodes with significant seromas which were punctured and episodes with significant seromas which were not punctured.

## Conclusion

A prompt acknowledgment of delayed methotrexate elimination and renal dysfunction is crucial to successfully manage potential methotrexate toxicity due to high MTX level in third space fluids.

We recommend punctures of periprosthetic seromas before or during MTX courses to avoid a possible contribution to third space effects, even though systemic effects on MTX kinetics, toxicities or treatment outcomes have to be evaluated in further studies [11, 12].
